# A Novel Machine Learning-Based ANFIS Calibrated RISS/GNSS Integration for Improved Navigation in Urban Environments

**DOI:** 10.3390/s24061985

**Published:** 2024-03-20

**Authors:** Ahmed E. Mahdi, Ahmed Azouz, Aboelmagd Noureldin, Ashraf Abosekeen

**Affiliations:** 1 Electrical Engineering Branch, Military Technical College (MTC), Cairo 11766, Egypt; ahmedelgibly@yahoo.com (A.E.M.); a.azouz@mtc.edu.eg (A.A.); 2 Electrical and Computer Engineering, Royal Military College of Canada (RMCC), Kingston, ON K7K 7B4, Canada; aboelmagd.noureldin@rmc.ca

**Keywords:** autonomous vehicle navigation, GNSS, INS, MEMS-IMU, machine learning, ANFIS, RISS, INS/GNSS integration

## Abstract

Autonomous vehicles (AVs) require accurate navigation, but the reliability of Global Navigation Satellite Systems (GNSS) can be degraded by signal blockage and multipath interference in urban areas. Therefore, a navigation system that integrates a calibrated Reduced Inertial Sensors System (RISS) with GNSS is proposed. The system employs a machine-learning-based Adaptive Neuro-Fuzzy Inference System (ANFIS) as a novel calibration technique to improve the accuracy and reliability of the RISS. The ANFIS-based RISS/GNSS integration provides a more precise navigation solution in such environments. The effectiveness of the proposed integration scheme was validated by conducting tests using real road trajectory and simulated GNSS outages ranging from 50 to 150 s. The results demonstrate a significant improvement in 2D position Root Mean Square Error (RMSE) of 43.8% and 28% compared to the traditional RISS/GNSS and the frequency modulated continuous wave (FMCW) Radar (Rad)/RISS/GNSS integrated navigation systems, respectively. Moreover, an improvement of 47.5% and 23.4% in 2D position maximum errors is achieved compared to the RISS/GNSS and the Rad/RISS/GNSS integrated navigation systems, respectively. These results reveal significant improvements in positioning accuracy, which is essential for safe and efficient navigation. The long-term stability of the proposed system makes it suitable for various navigation applications, particularly those requiring continuous and precise positioning information. The ANFIS-based approach used in the proposed system is extendable to other low-end IMUs, making it an attractive option for a wide range of applications.

## 1. Introduction

Autonomous vehicles (AVs) necessitate precise navigation systems to perform critical tasks such as enhanced driver vision, lane keeping, traffic flow guiding, and collision warning. Consequently, ensuring continuous and accurate navigation has become increasingly crucial for intelligent vehicles as well [1]. While Global Navigation Satellite Systems (GNSS) such as Global Positioning System (GPS) work as the primary source of precise navigation solutions, they are not without their challenges [2]. In urban environments, GNSS signals can be obstructed by tall buildings, affected by multipath interference, or even subjected to deliberate jamming, all of which can significantly impact navigation accuracy. To improve accuracy, AVs often employ a combination of sensors [3,4,5,6,7,8,9,10,11,12,13].

Inertial Navigation Systems (INS) offer continuous navigation solutions even in environments with degraded signal conditions. However, they are susceptible to errors over time due to their mathematical models, particularly in the case of micro-electromechanical system (MEMS)-based INS systems. To leverage the respective strengths of both INS and GNSS, integrating INS with GNSS has been widely adopted. This integration combines the continuous navigation capabilities of INS with the accuracy of GNSS, resulting in a more robust and reliable navigation solution that is less susceptible to errors and better suited for AVs [2,14,15,16,17,18].

An alternative to traditional INS for AVs is the reduced inertial sensor system (RISS), which employs a reduced number of sensors compared to the conventional six-sensor INS setup. By reducing the number of sensors, RISS mitigates the accumulation of errors caused by sensor imperfections. RISS leverages the vehicle’s odometer to calculate velocity, eliminating the need for a secondary integration process and thereby improving the accuracy of position calculation. Furthermore, RISS utilizes accelerometers instead of gyroscopes to calculate pitch and roll angles, as accelerometers are less prone to errors [19,20]. However, in the absence of reliable GNSS signals, the RISS navigation solution may experience drift over time, particularly when utilizing MEMS-based inertial sensors. Therefore, sensor fusion has emerged as a popular approach in recent years. This approach combines data from multiple sensors, including odometers, vision systems, Light Detection and Ranging (LiDAR), magnetometers, and Radio Detection and Ranging (Radar), to achieve a higher level of accuracy and robustness than what can be achieved with individual sensors alone. This is especially valuable in challenging environments such as urban areas or indoor settings [1,19,20,21,22,23,24].

However, the incorporation of additional sensors comes with associated costs, both in terms of increased system cost and complexity [23,25]. To overcome these challenges and improve navigation performance, researchers have increasingly relied on machine learning (ML) techniques, leveraging the extensive data obtained from inertial measurement unit (IMU) sensors to develop advanced navigation systems [26,27,28]. The utilization of ML techniques in navigation applications aims to exploit their capability to estimate nonlinear errors. ML algorithms can effectively learn the complex relationship between sensor measurements and the true position of the vehicle. Additionally, ML can identify and mitigate outliers present in sensor data due to noise, interference, or other factors. Traditional estimation methods such as the Kalman filter (KF), extended Kalman filter (EKF), unscented Kalman filter (UKF), invariant-EKF, or particle filter (PF) struggle to accurately model these errors. As a result, reducing the impact of nonlinear errors in IMU sensors enhances the performance of the RISS during GNSS outages [12,23,29,30,31,32,33].

### 1.1. Motivation

The paper addresses the challenges associated with the accumulation of errors in inertial navigation systems (INS) and the increased complexity and cost associated with integrating multiple aided sensors and systems [34,35]. Furthermore, each aided system or sensor has its own limitations, making it challenging to achieve optimal accuracy. To overcome these challenges, researchers have turned to machine learning (ML) techniques as a potential solution. ML offers the ability to accurately estimate and predict nonlinear errors, unlocking the full potential of inertial sensing applications. By combining ML with a Reduced Inertial Sensors System (RISS), the paper aims to improve navigation accuracy, particularly in challenging GNSS environments. The proposed approach seeks to overcome the limitations of traditional INS and the drawbacks of integrating multiple aided systems, while also considering cost and complexity constraints. To support the argument, previous studies by Li et al. [30,36] have shown promising results in employing ML techniques for navigation systems, and this paper aims to further demonstrate the benefits of ML in conjunction with RISS. By showcasing the effectiveness of this approach, the paper contributes to the advancement of navigation systems in scenarios where GNSS signals are unreliable or unavailable.

### 1.2. Objectives

This paper aims to enhance the accuracy of the 3D-RISS navigation solution during GNSS outages in urban environments. The proposed method utilizes ML-based ANFIS to estimate and predict linear/nonlinear errors in MEMS sensors to create a robust ML model for IMU measurements, improving the performance of low-end IMUs. Additionally, the paper presents a loosely coupled integrated navigation system that combines the 3D-RISS with ML-modified IMUs with a GNSS receiver. The effectiveness of the proposed system is demonstrated through tests conducted on a real trajectory with five different outage scenarios. The results showcase the superiority of the proposed system over the traditional RISS/GNSS integrated system, positioning it as a viable alternative to the Rad/RISS/GPS backup system that is introduced in [20,21,22] when GNSS signals are unavailable.

Finally, the main contributions of this paper can be summarized as follows:

1. Introducing a novel calibration method to enhance the performance of low-end IMU. It proposes the creation of a robust ML model using the ANFIS technique that predicts linear/nonlinear errors associated with its raw measurements.
2. Designing a pre-trained machine-learning-based 3D-RISS/GPS integrated navigation system that utilizes the ML-based ANFIS-modified IMU alongside the EKF to improve the integrated navigation solution, particularly in urban environments, without relying solely on GNSS signal availability and the need for online learning processes.
3. Evaluating the effectiveness of the proposed system through real road trajectory tests under different scenarios of GNSS outages, considering various durations and dynamics. The performance of the proposed system is extensively compared to the traditional RISS/GPS integrated navigation system and Rad/RISS/GPS system. The proposed system is introduced as an effective alternative to the Rad/RISS/GPS system for enhancing navigation accuracy during GNSS outages, achieving the aforementioned objective without inducing a substantial increase in the system’s overall cost or complexity.

### 1.3. Paper Organization

Section 1 provides an introduction to the challenges associated with traditional inertial navigation systems and the motivation for using machine learning techniques to improve navigation accuracy during GNSS outages. Section 2 presents related work that focuses on the utilization of artificial intelligence (AI) techniques in enhancing navigation solutions during GNSS outages and the use of radar as a navigation-aiding system. Section 3 describes the proposed methodology. Section 3.1 explains how the performance of a low-end IMU is leveraged using a machine-learning-based ANFIS technique. Section 3.2 presents the ML-based 3D-RISS mechanization, and Section 3.3 demonstrates the proposed ML-based RISS/GPS integrated navigation system scheme. Section 4 provides details on the experimental setup, including the systems and sensors used, along with their specifications. Section 5 presents the results of the proposed integrated navigation system compared to other systems. Finally, Section 6 summarizes the paper’s conclusions.

## 2. Related Work

In recent years, researchers have explored various AI techniques to improve navigation solutions during GNSS outages. Artificial neural networks (ANNs) have been used to enhance the performance of navigation systems during GNSS outages [37,38,39]. The introduced methodology primarily involves training the AI technique while the GNSS signal is available to estimate and predict INS errors. As a result, the AI technique can estimate the INS navigation solution during GNSS signal blockages, improving the system’s accuracy and reliability. Other AI techniques include fast orthogonal search (FOS) for nonlinear modeling of error estimation during the availability of GNSS signals [20]. However, in [40], FOS is introduced as an anti-jamming technique for GNSS signals, while supervised machine learning is stated in [41] as a detector of signal spoofing for GNSS.

Deep learning-based neural network (DNN) techniques have also been applied in various ways. In [42], a DNN technique is illustrated as a calibration methodology to denoise low-cost IMU gyroscopes and enhance attitude calculations. In [43], another deep learning-based NN technique is used to enhance calculated wheel speed measurements, thus improving position calculations in the RISS mechanization. Similarly, in [44], another deep learning-based NN is utilized to improve the INS solution in urban environments. The proposed AI algorithm is trained online during the presence of GNSS signals and enters the prediction mode during GNSS outages.

Fuzzy C-means (FCM) algorithm is utilized in [45] as a multi-sensor fusion technique, where FCM is employed to fuse data measured from the odometer and the radar as an aided system to improve forward speed calculations. An estimation algorithm is demonstrated in [8] to improve the accuracy of collected radar data. The algorithm mainly focuses on removing noise, outliers, and false objects. An optimization design between the fuzzy inference system (FIS) and the EKF is presented in [46], where FIS is used to adapt the initial knowledge of measurement and process covariance matrices to improve the estimation accuracy of the filter. The ANFIS is utilized to predict the navigation solution in [47], where the ANFIS model is trained using differential GNSS as a reference. The suggested methodology is validated using a public dataset (KITTI) over a trajectory lasting from 140 s to 400 s. The ANFIS technique has also been introduced in [48] as a real-time predictor for INS velocity and position errors during GNSS outages. However, the authors used navigational and tactical grades of the INS, not MEMS grade.

A diagnosis strategy for INS faults is provided in [49] by employing two enhanced GA-BP neural networks and the state Chi-square test. The state Chi-square test is used to identify the problem and extract feature data. The two BP neural networks, optimized by genetic algorithm, are trained to detect the fault and determine its type and magnitude. A comprehensive survey is provided in [30], which illustrates the role of AI techniques in improving the accuracy of inertial sensing applications. The survey discusses the advantages and drawbacks of each technique, as well as the general advantages and challenges of utilizing AI techniques in navigation applications.

On the other hand, radar uses electromagnetic waves to measure the distance, direction, and speed of objects. Radar waves are emitted from a transmitter and reflected back to the receiver when they hit an object. The time it takes for the waves to travel to and from the object is used to calculate the distance to the object. The direction of the object is determined by the angle at which the waves are reflected back to the receiver. The speed of the object is determined by the change in frequency of the waves as they reflect off the object. Radar is often used in combination with other navigation systems, such as GNSS and INS. Radar systems can provide information about objects that are not visible to other types of sensors, such as objects that are obscured by fog or rain. Additionally, radar systems can provide information about the speed and direction of objects, which can be important for collision avoidance. However, adding a radar system to a navigation system increases the system’s complexity and cost. Additionally, radar systems can be more difficult to calibrate and maintain. Moreover, a key constraint of radar is the processing time required for object distance, direction, and speed calculations [8,21,22,45].

According to the related work, all AI techniques have been trained using GNSS signals. Furthermore, there is a scarcity of using AI techniques to improve IMU sensor measurements. Therefore, this paper proposes a robust ML model to leverage the performance of a MEMS IMU. Moreover, the modified (pre-trained) sensors act as inputs to the 3D-RISS mechanization, which is then incorporated with GNSS receiver readings to provide a more robust navigation solution that is independent of GNSS signal availability.

## 3. Methodology

In scenarios of brief GNSS disruptions, the INS system is instrumental in producing navigation solutions by estimating states like position, velocity, and attitude, as discussed in previous sections. Modern navigation systems seamlessly integrate both INS and GNSS components, capitalizing on their complementary nature. During GNSS outages, the INS acts as the primary source of navigation information using the last known GNSS reading. However, it may generate a drifted solution due to errors in sensor angular rates and accelerations, particularly when using low-end IMUs. To address this, the proposed work employs a RISS to regulate the INS’s long-term drift.

The proposed work aims to enhance the precision of low-end IMU raw measurements by utilizing an ML-based ANFIS technique. By leveraging the improved sensors during integration with GNSS, the proposed approach strives to boost the accuracy of state prediction, resulting in an improved overall solution and restricting the INS’s drifted navigation solution during GNSS outages. The proposed approach employs a pre-trained IMU model, implying that no training process is necessary during the process. Both the training and model construction procedures are conducted in a previous stage using a high-end IMU unit as a pre-experiment calibration process.

### 3.1. Leveraging the Performance of a Low-End IMU Using ANFIS

**Figure 1:**
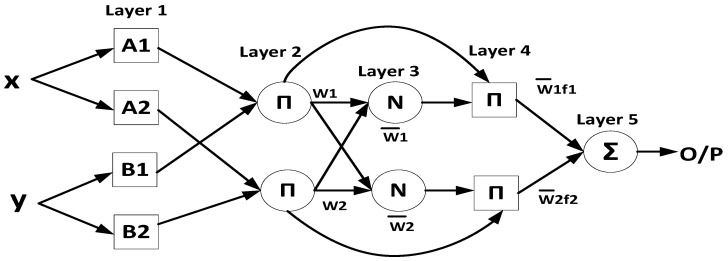
The basic structure of the ANFIS [50].

The proposed calibration technique incorporates an adaptive neuro-fuzzy inference system (ANFIS) as a calibration method for the low-end IMU. ANFIS combines the strengths of artificial neural network (ANN) and fuzzy inference system (FIS) techniques while compensating for their respective limitations. This integration enables the system to adapt through a self-organizing and self-learning process [50,51]. The ANFIS structure comprises five layers, each serving a distinct purpose. In the first layer, the membership function (MF) is applied to assign crisp inputs to each node, indicating the degree of match between the input and linguistic label. The second layer multiplies the input signals at each node to determine the weights of the rules, also known as firing strength. The third layer normalizes the weights of each rule by computing the ratio of each rule’s weight to the sum of all rule weights. In the fourth layer, the normalized weights of each rule are multiplied by the output of the second layer. Finally, the fifth layer sums all the incoming signals to compute the overall output. The structure of the ANFIS system is illustrated in Figure 1.

**Figure 2:**
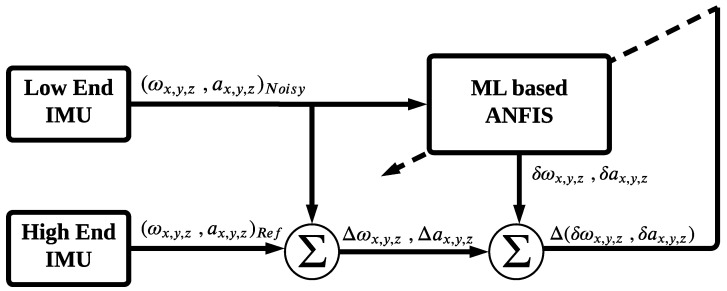
The training phase for the ML ANFIS model.

**Figure 3:**
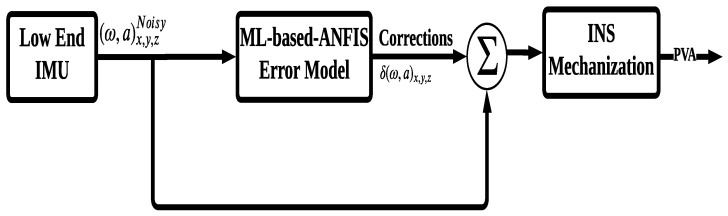
The validation phase of the ML ANFIS model.

The generation of the modified IMU involves two phases: the training phase and the validation phase, as shown in Figure 2 and Figure 3, respectively. During the training phase, the ANFIS technique utilizes input from the raw measurements of both the low-end IMU (denoted by “x”) and the high-end IMU (denoted by “y”), as illustrated in Figure 1, to train the ANFIS system and build the model. The primary objective of this stage is to generate a model capable of mitigating both linear and nonlinear errors associated with the low-end IMU’s raw measurements, as depicted in Figure 2. Consequently, the generated model is applied to this specific low-end IMU to provide a more accurate navigation solution throughout subsequent trajectories.

The ML-based ANFIS model is trained using the measurement errors $\delta {\left(\omega ,a\right)}_{x,y,z}$ associated with the IMU’s angular rates and accelerations, as depicted in Equation (1).

(1) $$\begin{array}{c} \delta {\left(\omega ,a\right)}_{x,y,z}={\left(\omega ,a\right)}_{x,y,z}^{Noisy}-{\left(\omega ,a\right)}_{x,y,z}^{Ref} \end{array}$$

where ${\left(\omega ,a\right)}_{x,y,z}^{Noisy}$ denotes the angular rates and accelerations from the low-end IMU in the (x,y,z) directions, respectively, while ${\left(\omega ,a\right)}_{x,y,z}^{Ref}$ represents the high-end IMU angular rates and accelerations in the (x,y,z) directions, respectively.

The selected model $ANFIS_{model}$ is the one that provides a minimum mean square error (MMSE) and the model selection criteria is shown in Equation (2).

(2) $$ANFIS_{model}=arg_{min}\left(\frac{1}{n}\sum_{n=1}^{n}\delta {\left(\omega ,a\right)}_{x,y,z}^{2}\right)$$

The second stage of the proposed calibration technique is the validation phase, as depicted in Figure 3. In this phase, the previously generated model is applied to the low-end IMU to mitigate associated errors. Additionally, the high-end IMU measurements are used for comparison purposes to evaluate the performance of the ML-based ANFIS model. The outcome of this phase is a modified/calibrated IMU that surpasses the capabilities of the original low-end IMU. Finally, the produced modified IMU acts as input to the INS mechanization, which can operate independently or be integrated with GNSS readings to provide a more reliable and accurate navigation solution.

To accomplish this objective, the proposed calibration technique can be conducted offline in a laboratory using a turntable or by conducting a short trajectory involving both stationary and in-motion dynamics as a pre-processing step. Therefore, a short trajectory is performed, and half of the collected data are used for the training phase, while the remaining half are used for the validation phase.

To validate the effectiveness of the proposed algorithm, a 3D-RISS system is employed instead of the complete INS due to the specific application nature of autonomous land vehicle navigation. The use of the modified 3D-RISS system provides a suitable platform for evaluating the effectiveness of the proposed approach in enhancing INS navigation solutions during GNSS outages, particularly in challenging environments. By utilizing the ML-based ANFIS technique to modify the low-end IMU’s sensors, the modified 3D-RISS system can offer more accurate and reliable navigation solutions compared to traditional INS systems.

### 3.2. ML-Based 3D-RISS

**Figure 4:**
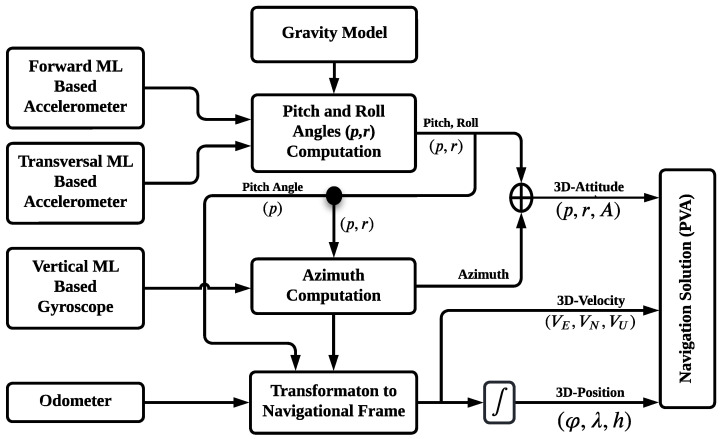
The ML-based RISS block diagram.

Compared to the traditional INS, the 3D-RISS offers a complete navigation solution, including 3D position, velocity, and attitude, with a reduced number of sensors. As mentioned in the previous subsection, the proposed approach utilizes a modified 3D-RISS, which is composed of four sensors. Three of these sensors (vertical gyroscope and longitudinal and transversal accelerometers) are modeled using the ML-based ANFIS technique proposed in this work, while the fourth sensor is an odometer. A block diagram of the ML-based 3D-RISS is shown in Figure 4.

As depicted in the ML-based 3D-RISS block diagram, its mechanization can be expressed as detailed below.

#### 3.2.1. Pitch and Roll Angle Calculation

The pitch angle can be calculated utilizing the odometer, transversal accelerometer, and gravity. It is important to note that gravity varies based on the object’s latitude and height. The modified pitch angle can be estimated as in Equation (3).

(3) $$p=sin^{-1}\left(\frac{f_{y}^{ML}-a_{od}}{g}\right)$$

where *p* is the modified pitch angle, $f_{y}^{ML}$ is the forward accelerometer’s specific force after applying the ML technique, $a_{od}$ is the odometer’s forward acceleration, and *g* is the gravity. Integrating the gravity model into the 3D-RISS mechanization can significantly enhance the accuracy of pitch angle calculations, as opposed to using a fixed value for gravity. In other words, the gravity is determined as in Equation (4) [19].

(4) $$g=g_{WGS0}\frac{1+g_{WGS1}sin\left(\varphi \right)}{\sqrt{1-\;E^{2}{sin}^{2}(\varphi })}-\left[3.0877-0.0044\;{sin}^{2}\left(\varphi \right)\right]10^{-6}h+0.072\times 10^{-12}$$

where the equatorial gravity $g_{WGS0}=9.78032677$ m/s^2^, the gravity formula constant is $g_{WGS1}=0.00193185138639$ m/s^2^, and the second eccentricity is $E^{2}=0.0818191908426$.

Depending on the odometer, transversal accelerometer, and the vertical gyroscope, the modified roll angle can be estimated as in Equation (5).

(5) $$r=-sin^{-1}\left(\frac{f_{x}^{ML}-v_{od}\left(\omega_{z}^{ML}-b_{z}\right)}{gcos(p)}\right)$$

where *r* is the modified roll angle, $f_{x}^{ML}$ is the ML-based transversal accelerometer’s specific force, $\omega_{z}^{ML}$ is the ML-based angular rate, $b_{z}$ is the gyroscope’s bias, and $v_{od}$ is the odometer’s forward speed.

#### 3.2.2. Azimuth Rate

The modified azimuth rate $\dot{A}$ calculation is determined from the gyroscope’s angular rate, the Earth’s rotation rate, and meridian radius of curvature of the Earth, as in Equation (6).

(6) $$\dot{A}=-\left(\omega_{z}^{ML}-b_{z}-\omega^{ie}sin\left(\varphi \right)-\frac{v^{e}tan\left(\varphi \right)}{R_{N}+h}\right)$$

where $\omega^{ie}$ is the Earth’s rotation rate, $v_{e}$ is the east velocity, and $R_{N}$ is the meridian radius.

#### 3.2.3. Velocity and Position Calculations

The 3D velocity components are determined from the odometer’s forward speed, as in Equation (7).

(7) $$\left[\begin{array}{c} v^{e}\\ v^{n}\\ v^{u} \end{array}\right]=\left[\begin{array}{c} v_{od}sin\left(A\right)cos\left(p\right)\\ v_{od}cos\left(A\right)cos\left(p\right)\\ v_{od}sin\left(p\right) \end{array}\right]$$

where $v^{e}$, $v^{n}$, and $v^{u}$ are the east, north, and up velocities, respectively.

The 3D position components are derived from the 3D velocity components, as in Equation (8):

(8) $$\left[\begin{array}{c} \dot{\varphi }\\ \dot{\lambda }\\ \dot{h} \end{array}\right]=\left[\begin{array}{c} \frac{v^{n}}{R_{N}+h}\;\\ \frac{v^{e}}{\left(R_{M}+h\right)cos\left(\varphi \right)}\;\\ v^{u} \end{array}\right]$$

where $\dot{\varphi },$$\dot{\lambda }$, and $\dot{h}$ are the latitude, longitude, and altitude rates, respectively, and $R_{M}$ is the normal radius of curvature of the Earth’s ellipsoid. The pitch angle, roll angle, and azimuth rate in the RISS mechanization are primarily dependent on the ML-based gyroscope and accelerometers, as shown in the previous equations. Given this dependence, an integration scheme is proposed between the modified RISS mechanization and GPS to further enhance the accuracy of the navigation solution during GPS outages.

To show the effectiveness of using the ANFIS as a calibration technique, cumulative distribution function (CDF) plots [52,53] are presented for the utilized measurements that work as a control input to the RISS mechanization ($\delta f_{x},\delta f_{y},and\;\delta w_{z}$).

The CDF for errors signifies the probability that a random variable describing errors is less than or equal to a given point. In the context of errors, including measurement errors or prediction errors, the CDF provides valuable insights into the likelihood of observing errors within a specific range. If (X) is a random variable representing errors, the CDF (F(x)) is mathematically defined as (F(x)=P(X≤x)), where (F(x)) represents the probability that the error is less than or equal to (x). The CDF exhibits non-decreasing behavior within the interval ([0,−∞]), approaching 1 as (x) tends to (∞). In practical applications, the CDF finds extensive use in analyzing error distributions across various domains, such as statistics, machine learning, and signal processing. Its application aids in comprehending the behavior of errors and facilitates the assessment of model or system performance by quantifying the likelihood of different error magnitudes [54].

**Figure 5:**
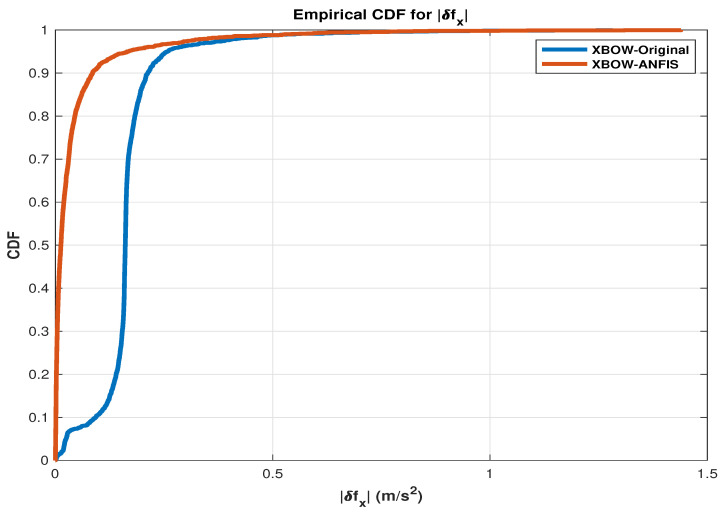
A CDF comparison plot for the forward accelerometer error.

**Figure 6:**
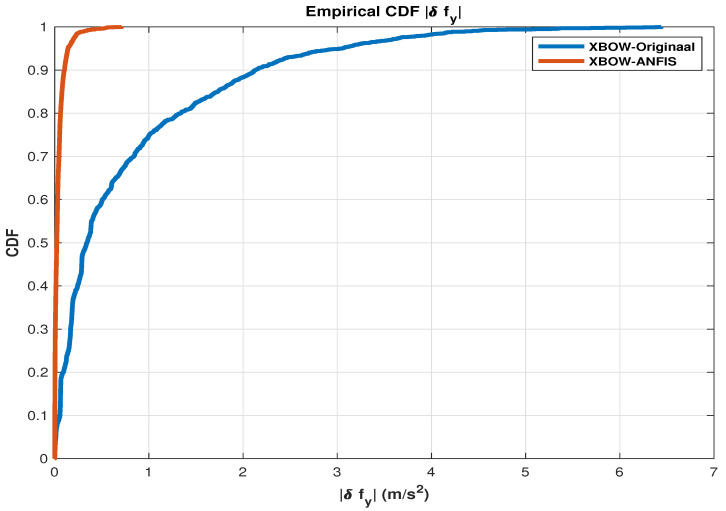
A CDF comparison plot for the transversal accelerometer error.

**Figure 7:**
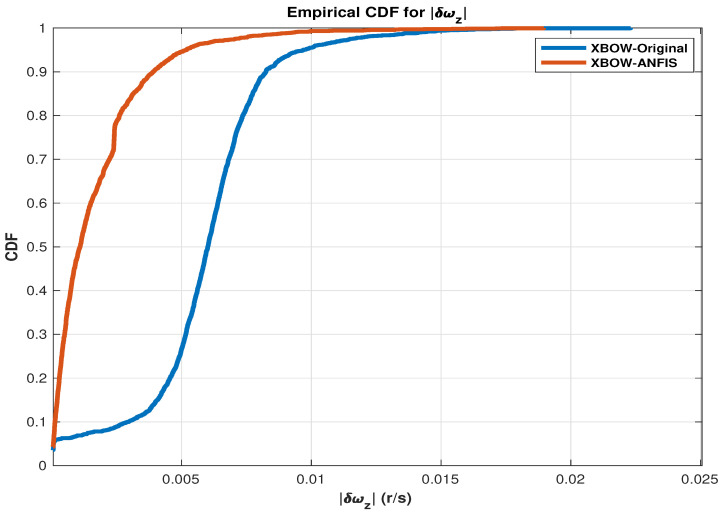
A CDF comparison plot for the vertical gyroscope error.

The presented CDF plots show the improvement of the IMU measurements after applying the ANFIS compared to the original IMU measurements, as shown in the following Figure 5, Figure 6 and Figure 7.

As shown in the presented CDF comparison, a significant improvement of the inertial sensors measurements is achieved when applying the ML-based ANFIS algorithm. These results lead to an improved navigation solution, which reduces the error growth problem of the RISS, especially during the GNSS outages.

### 3.3. ML-Based RISS/GPS Integration

**Figure 8:**
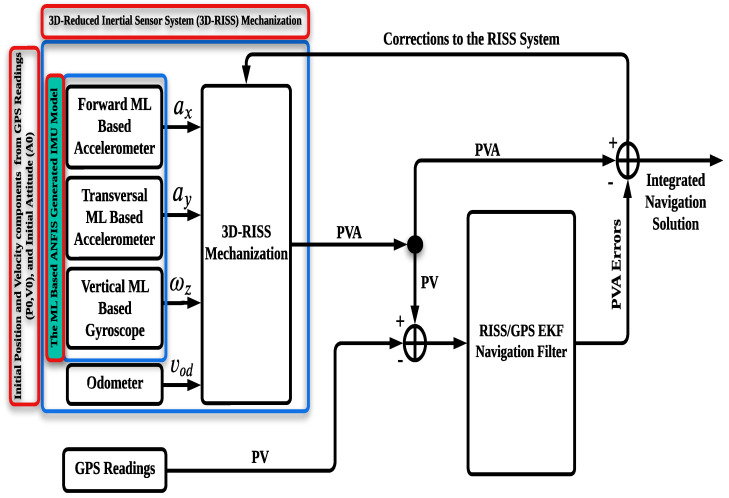
The ML-based RISS/GPS system block diagram.

The ML-based RISS/GPS integrated navigation system composed of 3D-RISS modified by the ML-based ANFIS technique and a GPS receiver in a loosely coupled integration scheme is illustrated in Figure 8.

The EKF is introduced in our work as the navigation filter to rectify the RISS errors. As can be seen, it utilized the Taylor expansion as a linearizing technique by taking only the first order of the expansion. So, the system error patterns composed of the nine states (position and velocity components, azimuth, and the bias drift of the accelerometers and the gyroscope) are shown in Equation (9).

(9) $$\delta x={\left[\begin{array}{c} \delta \varphi ,\;\delta \lambda ,\;\delta h,\;\delta v^{e},\;\delta v^{n},\;\delta v^{u},\;\delta A,\;\delta a_{od},\;\delta b_{z} \end{array}\right]}^{T}$$

where δφ, δλ, and δh are the position error components (latitude, longitude, and altitude), respectively. $\delta v^{e}$, $\delta v^{n}$, $\delta v^{u}$ are the velocity error components (east, north, and up), respectively. δA is the azimuth error component, $\delta a_{od}$ is the accelerometer’s bias drift determined from the odometer calculations, and $\delta b_{z}$ is the gyroscope’s bias drift.

Therefore, the system model is calculated according to Equation (10).

(10) $$\delta x_{k}=\Phi_{k-1}\delta x_{k-1}+G_{k}w_{k}$$

where $\delta x_{k}$ is the epoch (*K*)’s error vector, $\Phi_{k-1}$ is the previous epoch (k−1)’s transition matrix, $G_{k}$ is the noise distribution matrix, and $w_{k}$ is the process noise - White Gaussian Noise (WGN) with zero-mean and covariance $Q_{k}$. The linearized system model and its design matrix F are typical, as explained in [19].

Additionally, the measurement model is represented by Equation (11).

(11) $$\delta z_{k}=H\delta x_{k}+\varepsilon$$

where $\delta z_{k}$ is the measurement vector, *H* is measurement’s design matrix, and ε is the GPS associated noise.

Thus, the measurement vector is as shown in Equation (12) and the design matrix is as shown in Equation (13), while the term of the noise is treated as white noise determined by the R matrix, as in Equation (14).

(12) $$\delta z_{k}=\left[\begin{array}{c} \varphi_{RISS(Mod)}-\varphi_{GPS}\\ \lambda_{RISS(Mod)}-\lambda_{GPS}\\ h_{RISS(Mod)}-h_{GPS}\\ v_{RISS(Mod)}^{e}-v_{GPS}^{e}\\ v_{RISS(Mod)}^{n}-v_{GPS}^{n}\\ v_{RISS(Mod)}^{u}-v_{GPS}^{u} \end{array}\right]$$

(13) $$H=\left[\begin{array}{ccccccccc} 1 & 0 & 0 & 0 & 0 & 0 & 0 & 0 & 0\\ 0 & 1 & 0 & 0 & 0 & 0 & 0 & 0 & 0\\ 0 & 0 & 1 & 0 & 0 & 0 & 0 & 0 & 0\\ 0 & 0 & 0 & 1 & 0 & 0 & 0 & 0 & 0\\ 0 & 0 & 0 & 0 & 1 & 0 & 0 & 0 & 0\\ 0 & 0 & 0 & 0 & 0 & 1 & 0 & 0 & 0 \end{array}\right]$$

(14) $$R=\left[\begin{array}{cccccc} \sigma_{\varphi_{GPS}}^{2} & 0 & 0 & 0 & 0 & 0\\ 0 & \sigma_{\lambda_{GPS}}^{2} & 0 & 0 & 0 & 0\\ 0 & 0 & \sigma_{h_{GPS}}^{2} & 0 & 0 & 0\\ 0 & 0 & 0 & \sigma_{v_{GPS}^{e}}^{2} & 0 & 0\\ 0 & 0 & 0 & 0 & \sigma_{v_{GPS}^{n}}^{2} & 0\\ 0 & 0 & 0 & 0 & 0 & \sigma_{v_{GPS}^{u}}^{2} \end{array}\right]$$

**Figure 9:**
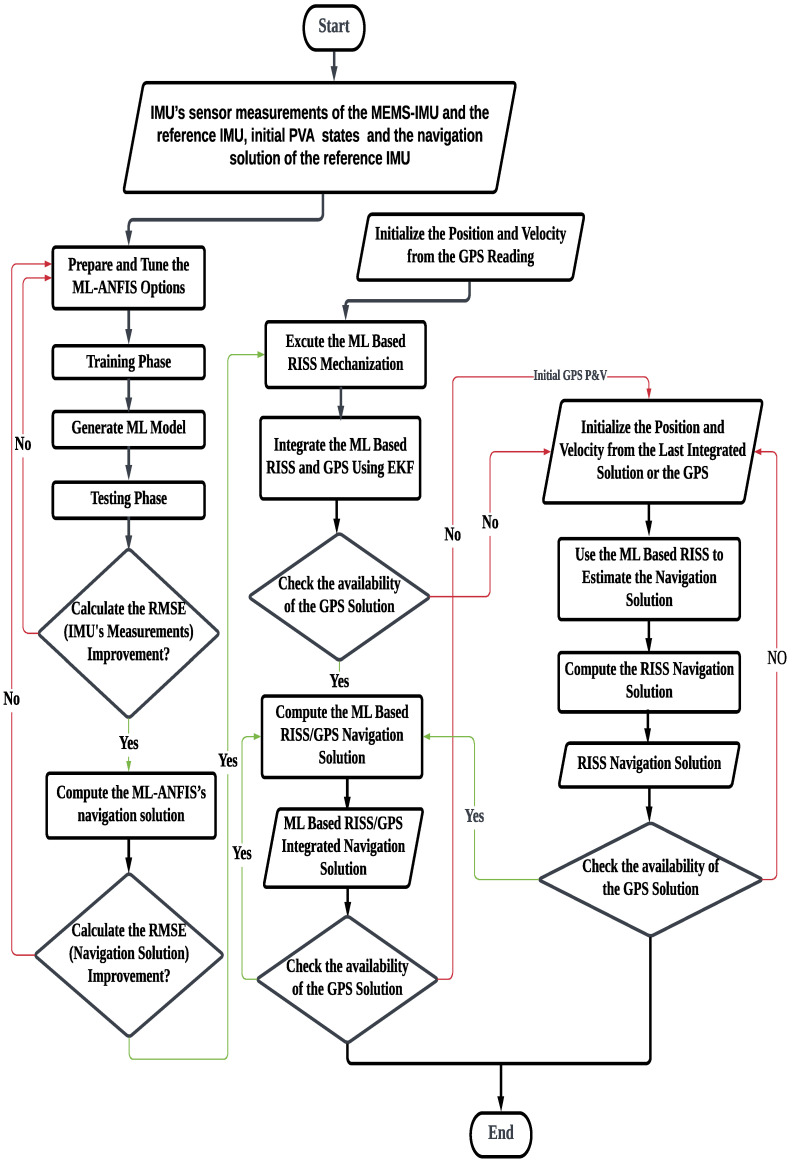
The overall flowchart of the proposed methodology.

Alternatively, the proposed approach leverages the power of ML techniques to estimate the linear and nonlinear errors of the IMU and reduce the sensors’ error growth over time. Furthermore, the ML model is also utilized in the RISS/GPS integration scheme to further improve the accuracy of the navigation solution during GPS outages. Therefore, a flowchart depicting the overall algorithm of the proposed methodology is presented in Figure 9.

## 4. Experimental Setup

**Figure 10:**
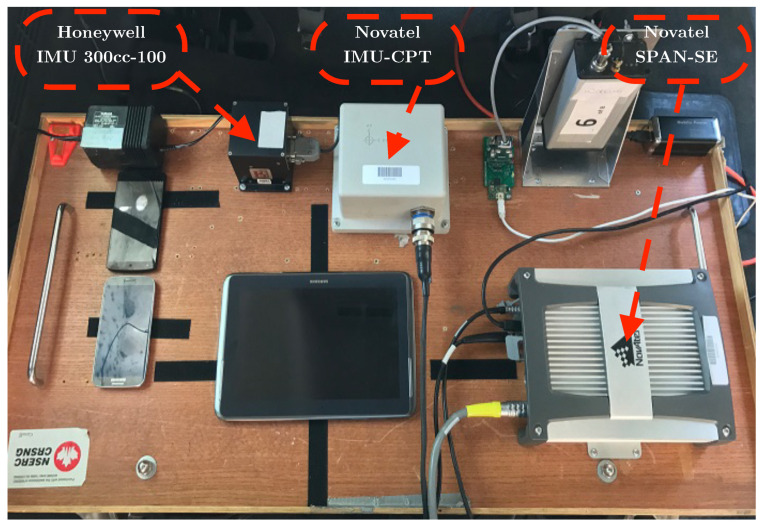
Interior test-bed showing the units involved in the experiment.

To validate the performance of the proposed navigation system, an experimental study was conducted using an actual road trajectory in the downtown area of the city of Kingston, ON, Canada. The study involved mounting a test-bed on the test vehicle, which consists of a number of sensors capable of collecting data about the vehicle’s position, orientation, and speed, as shown in Figure 10.

The van’s axes were aligned with the interior test-bed installation to ensure accurate measurement of the vehicle’s movement. Furthermore, the test-bed was securely and rigidly fixed in the back seat area using a typical seat chassis, ensuring that the collected data were reliable and accurate. The two IMUs used in this study were the XBOW IMU300CC, a MEMS grade IMU from Honeywell (Charlotte, NC, USA), and IMU-CPT, a high-end IMU from Hexagon/Novatel (Calgary, AB, Canada). The specifications of these IMUs are illustrated in Table 1.

The GNSS receiver used in the proposed integration scheme was the Novatel dual-frequency GNSS receiver (SPAN-OEM4 or SPAN-SE) with an output rate of 1 Hz. The IMU-CPT was used in conjunction with the GNSS receiver to form a tightly coupled GNSS/IMU system that provides highly accurate navigation solutions used as a reference. Therefore, the navigation solution generated by the proposed integration scheme was compared to the reference solution and to the noisy one to validate the effectiveness of the proposed approach.

In addition to the GNSS/IMU system, the radar development kit (RDK)(RK1001K/00) from Sivers Semiconductors (Kista, Stochholm, Sweden) was utilized for comparison purposes. The RDK operates at a frequency of 24.5 GHz and can detect objects with a maximum speed of 215 km/h. It can also measure the speed of objects and has a 3 dB beamwidth angle of 9.5°.

### 4.1. Data Collection

During the experimental study, data were collected using the mounted sensors and the test vehicle moving along a predefined road trajectory. The collected data included GNSS measurements, IMU readings, and radar data. The synchronized data from these sensors were then used for evaluating the performance of the proposed navigation system.

### 4.2. Reference System

To establish a ground truth reference for the experimental study, the tightly coupled GNSS/IMU system, consisting of the Novatel GNSS receiver and IMU-CPT, was considered. This reference system provided accurate and reliable navigation solutions for comparison with the proposed navigation system’s output.

### 4.3. Comparison Metrics

The performance of the proposed navigation system was assessed using various metrics, including position accuracy, orientation accuracy, and speed accuracy. These metrics were calculated by comparing the navigation solution generated by the proposed system with the reference GNSS/IMU system. The evaluation metrics aimed to quantify the system’s effectiveness in providing accurate navigation solutions in real-world scenarios.

## 5. Results and Discussion

**Figure 11:**
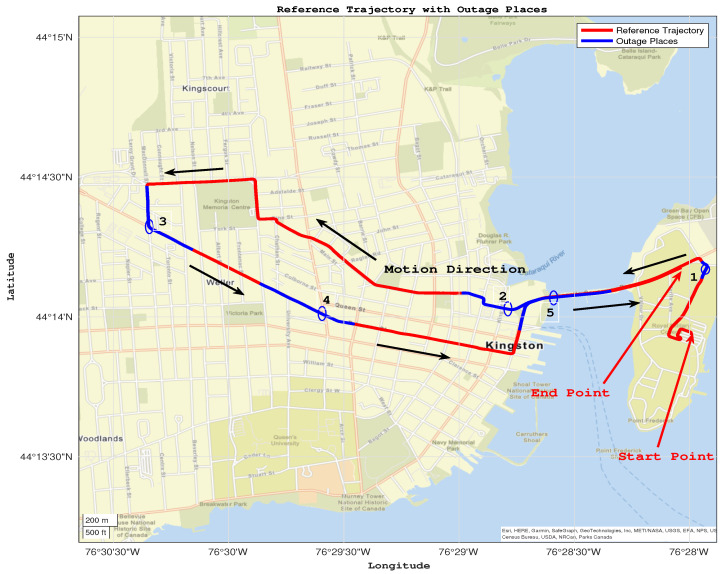
The reference trajectory with GNSS outage places and their numbers.

The proposed navigation system was tested on an actual road trajectory in the downtown area of Kingston, ON, Canada. The trajectory included five different GPS outages of varying lengths, ranging from 50 to 150 s. The red line in the trajectory map indicates the path taken, with the start and end points marked. The GPS outages are shown in blue, with circles indicating their midpoint, as shown in Figure 11.

The GPS outages were selected to include a variety of driving scenarios, including turns, sequential turns, stops, crossing intersections, and straight driving. Additionally, the outages were conducted at different speeds, adding to the complexity of the driving conditions. The use of a real-world road trajectory with diverse driving conditions and GPS outages provides a platform for evaluating the proposed approach’s effectiveness in enhancing navigation accuracy during GPS outages.

**Figure 12:**
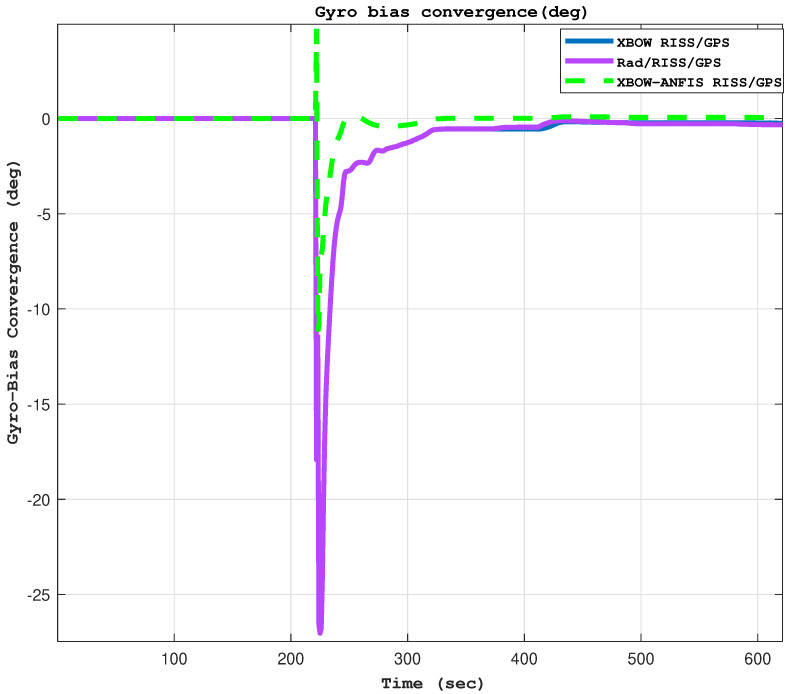
The gyroscope bias convergence time comparison.

Figure 12 shows a comparison of the gyroscope bias convergence between the XBOW RISS/GPS, Rad/RISS/GPS, and XBOW-ANFIS RISS/GPS systems. The results clearly demonstrate that the XBOW-ANFIS RISS/GPS system has a significantly lower gyroscope bias drift than the other two systems. This is attributed to the ANFIS algorithm’s ability to effectively compensate for the gyroscope drift, resulting in faster convergence time and a more accurate azimuth estimate.

**Figure 13:**
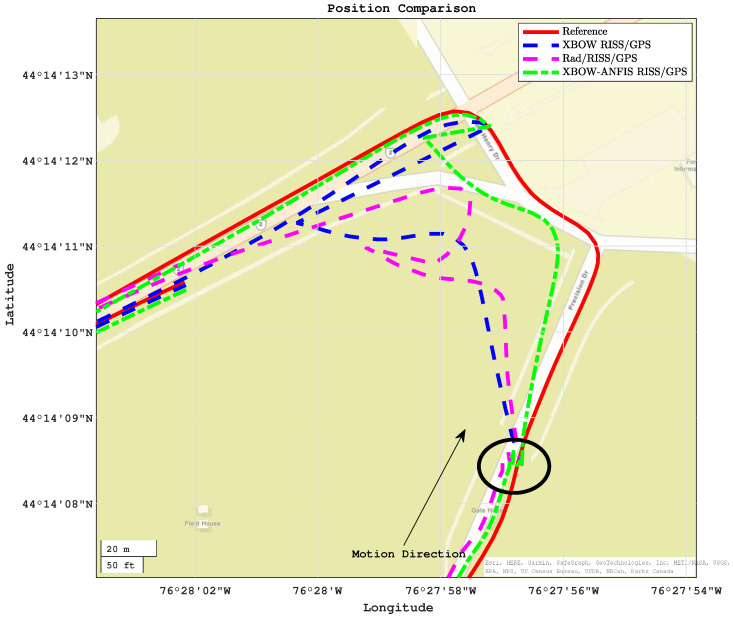
Positioning performance during outage 1.

**Figure 14:**
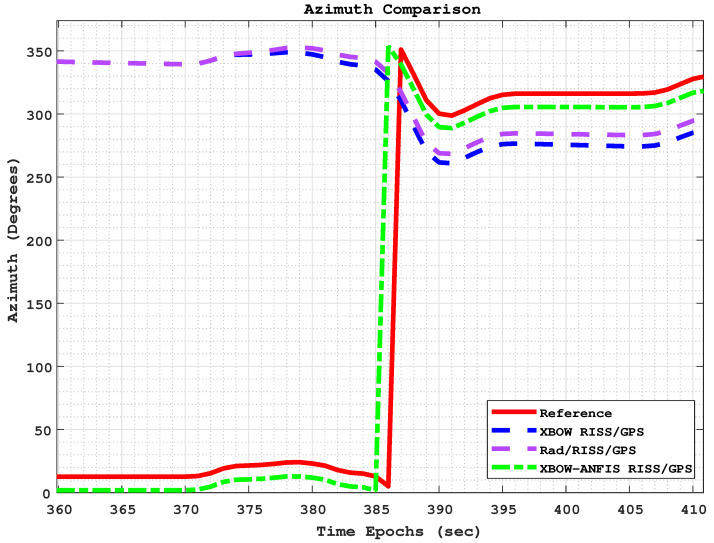
The azimuth comparison through outage 1.

Figure 13 and Figure 14 show the comparison of positioning and azimuth estimates, respectively, during the first GPS outage, which lasted for 50 s and included two consecutive turns after a stop sign. This driving scenario induced a lot of noise in the GPS signal, making it challenging for the XBOW RISS/GPS and Rad/RISS/GPS systems to accurately estimate position and azimuth. However, the proposed modified system was able to maintain a more accurate azimuth estimate, which enabled it to accurately estimate the vehicle’s position even through the turns.

In this driving scenario, the maximum 2D position error of the XBOW RISS/GPS system reached 68.7 m, while the maximum error for the Rad/RISS/GPS system was 54.8 m. In contrast, the proposed modified system demonstrated a significant improvement in position accuracy, with a maximum error of 25.8 m. This represents a 62.4% and 53% improvement over the XBOW RISS/GPS and Rad/RISS/GPS systems, respectively.

**Figure 15:**
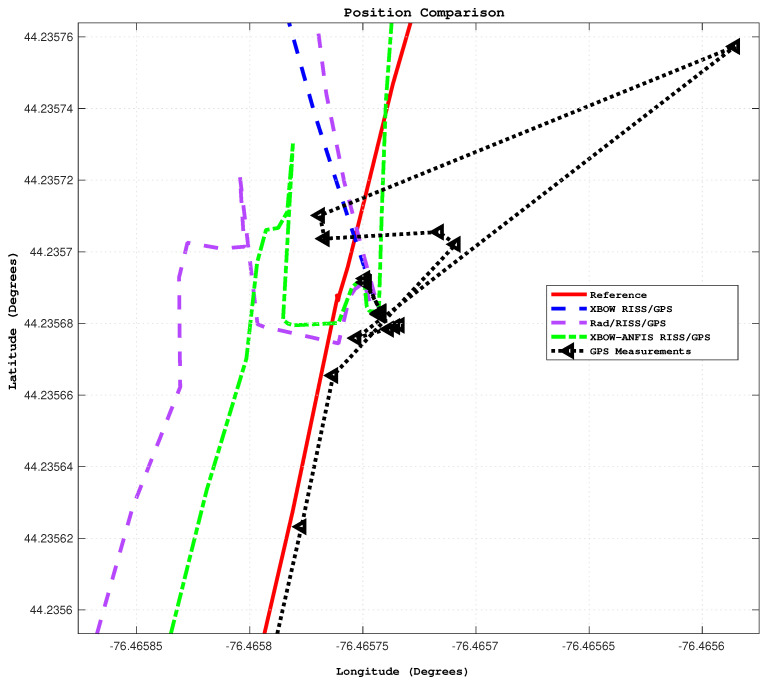
A zoomed– in trajectory segment before outage 1.

Additionally, it is important to note that the solutions of all systems were already diverging before the start of the outage due to the poor GPS updates. Specifically, the GPS position was not reliable right before the start of outage 1, which could be considered a prolonged outage. However, the proposed ANFIS-based solution was able to sustain reliable 2D position accuracy with root mean square error (RMSE) of 15 m, whereas the RISS/GPS and Rad/RISS/GPS systems achieved 45 m and 36 m, respectively. This outcome highlights the ability of the proposed system to limit error growth before and during GPS outages. A zoomed-in trajectory segment (circled in Figure 13) demonstrating the system’s performance just before the outage including the GPS measurements is illustrated in Figure 15.

During the third GPS outage, which lasted for 60 s, the vehicle was making a slight left turn while traveling at an average speed of 50–60 km/h. The proposed integration scheme successfully reduced the maximum 2D position error from 23.5 m for the XBOW RISS/GPS system and 19.9 m for the Rad/RISS/GPS system to 15.8 m, representing an improvement of 32.7% over the XBOW RISS/GPS system and 20.6% over the Rad/RISS/GPS system.

**Figure 16:**
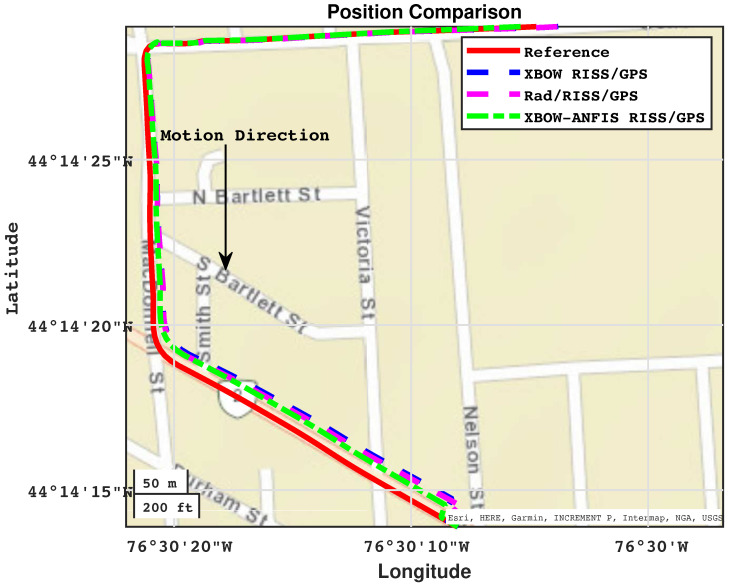
Positioning performance during outage 3.

**Figure 17:**
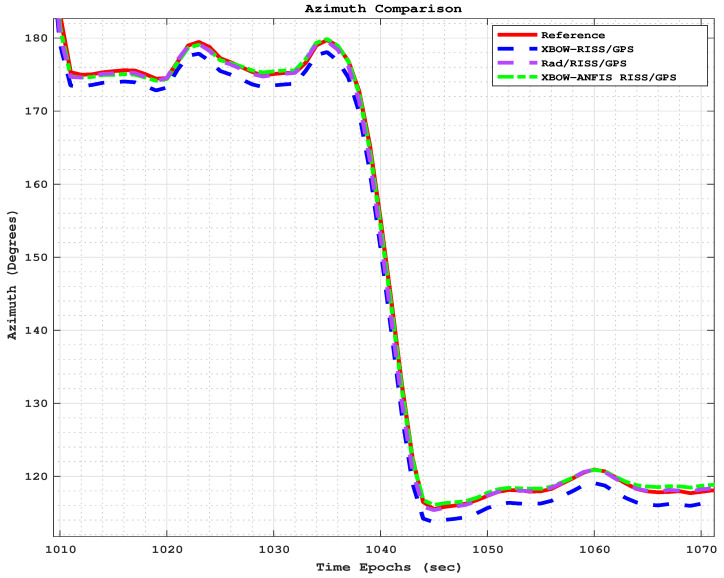
The azimuth comparison through outage 3.

Figure 16 and Figure 17 show the positioning and azimuth comparison, respectively, during the third GPS outage. The results show that the proposed modified system was able to maintain accurate positioning and azimuth estimates, even during the challenging driving scenario of a left turn at high speed.

**Figure 18:**
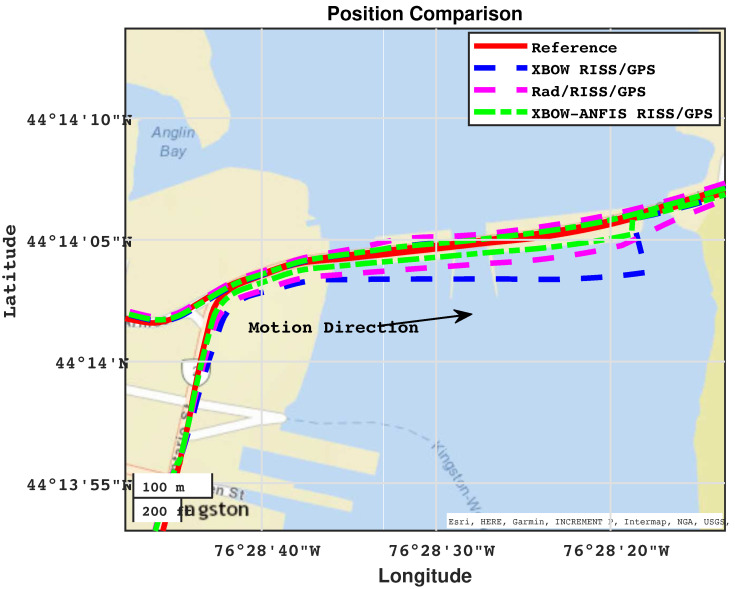
Positioning performance during outage 5.

**Figure 19:**
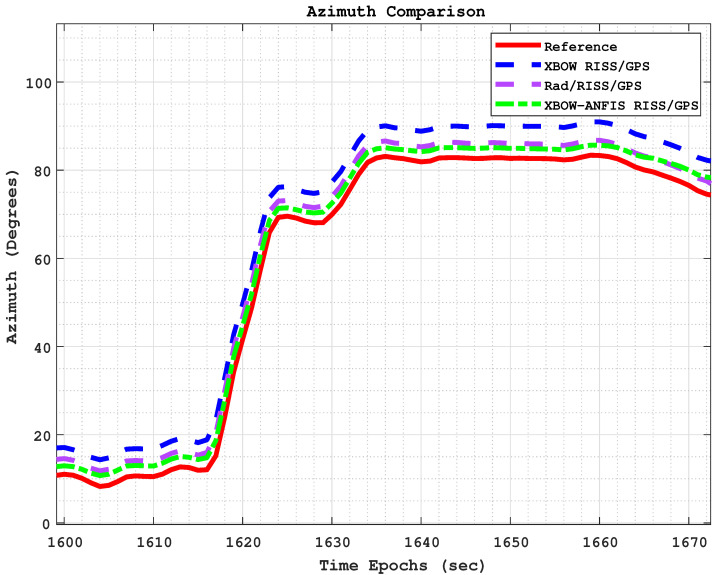
The azimuth comparison through outage 5.

Figure 18 and Figure 19 show the positioning and azimuth comparison, respectively, during the fifth GPS outage. This outage lasted for 70 s; the vehicle was crossing Kingston Bridge at a slow speed of 16 km/h. The driving scenario included a gathering between left and right turns with no stops. Once again, the proposed integration scheme demonstrated its effectiveness by reducing the maximum 2D position error from 67.5 m for the XBOW RISS/GPS system and 32.5 m for the Rad/RISS/GPS system to 18.8 m. This represents a significant improvement of 72.1% over the XBOW RISS/GPS system and 42.1% over the Rad/RISS/GPS system.

**Figure 20:**
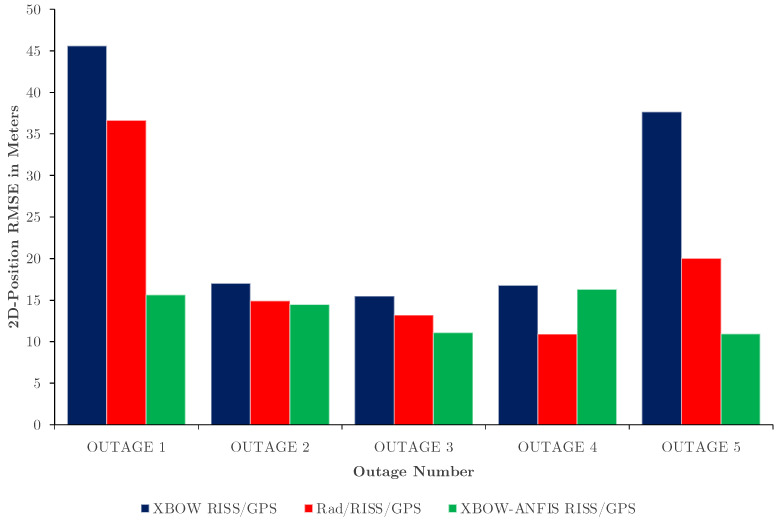
2D position RMSE comparison.

Table 2 presents the 2D and 3D position RMSE as an essential standard for evaluating the proposed integration scheme, while Figure 20 demonstrates a bar graph of the 2D position RMSE comparison.

The results show that the proposed integration scheme XBOW-ANFIS RISS/GPS has a significantly lower average 2D/3D position RMSE compared to the other two systems, XBOW RISS/GPS and Rad/RISS/GPS. The average 2D position RMSE is reduced from 26.5 m in the XBOW RISS/GPS system and 19.14 m in the Rad/RISS/GPS system to 13.67 m in the XBOW-ANFIS RISS/GPS system, with an improvement of 43.8% and 28.2%, respectively.

Furthermore, the selected outages were designed to encompass a range of dynamics that a vehicle may encounter in challenging GPS environments. This includes scenarios involving straight driving (GPS fourth outage), which could be a limitation of the proposed method compared to the Radar/RISS/GPS solution. Since the vehicle was moving strictly along a straight line during this outage, the Radar/RISS/GPS solution was able to show some advantages, resulting in better positioning accuracy of 10.93 m, instead of 16.28 m for the ANFIS-based solution. In this scenario, the primary sensor used for obtaining the navigation solution in both our proposed system and the traditional RISS/GPS system is the odometer. However, it is important to note that the ML-based ANFIS does not modify the odometer readings, and there are no significant changes in the azimuth calculations that can be enhanced by our system. Therefore, the improvement of this particular outage was less than the other outages.

However, compared to the traditional RISS/GPS, the proposed method still outperforms it for all GPS outages. Additionally, during the remaining outages, the proposed method yields a superior positioning solution, as indicated in Table 2, Table 3 and Table 4. By incorporating such diverse situations, we aimed to thoroughly evaluate the method’s performance in terms of robustness and limitations.

**Figure 21:**
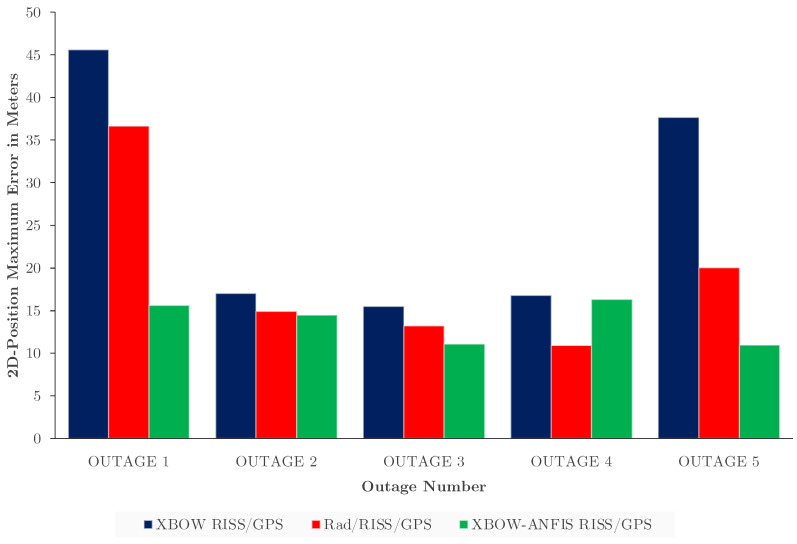
2D position max error comparison.

The proposed integration scheme, XBOW-ANFIS RISS/GPS, also has a significantly lower maximum position error compared to the other two systems, XBOW RISS/GPS and Rad/RISS/GPS. This means that the proposed integration scheme is able to estimate positions with a much smaller margin of error than the other two systems. Table 3 presents the 2D and 3D position maximum error, while Figure 21 illustrates the 2D position maximum error in a bar graph. These results demonstrate a significant improvement when applying the proposed XBOW-ANFIS RISS/GPS compared to the XBOW RISS/GPS and Rad/RISS/GPS. The maximum 2D position error is reduced from 44.8 m in the XBOW RISS/GPS system and 30.70 m in the Rad/RISS/GPS system to 23.5 m in the XBOW-ANFIS RISS/GPS system. This represents a 47.5% and 23.4% improvement, respectively. It is worth noting that the Rad/RISS/GPS system performed the best during the fourth GPS outage, as previously mentioned, due to the outage’s dynamics.

**Figure 22:**
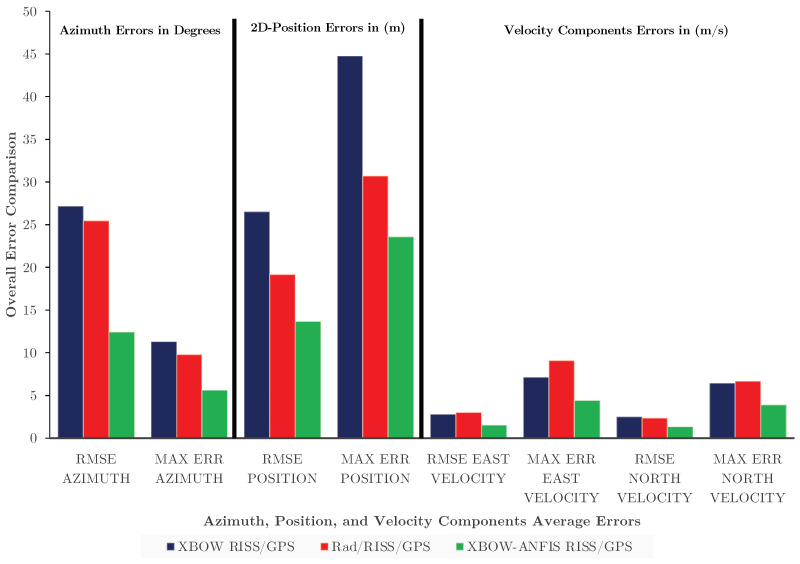
Overall performance comparison.

The results shown in Table 4 and Figure 22 present an overall performance comparison in terms of average RMSE and maximum azimuth error, 2D position, east velocity, and north velocity for the three systems.

The XBOW-ANFIS RISS/GPS system outperforms the other two systems, with the minimum average RMSE and maximum error for all four metrics. This means that the XBOW-ANFIS RISS/GPS system provides the most accurate estimation of azimuth, position, east velocity, and north velocity. The improvement in accuracy with the XBOW-ANFIS RISS/GPS system is most significant for azimuth estimation. The RMSE azimuth is improved by 54.23% compared to the XBOW RISS/GPS system and 51.14% compared to the Rad/RISS/GPS system. Similarly, the maximum error azimuth is reduced by 50.26% compared to the XBOW RISS/GPS system and by 42.65% compared to the Rad/RISS/GPS system.

However, the improved accuracy in azimuth estimation with the XBOW-ANFIS RISS/GPS system also leads to improved position estimation, resulting in the minimum average RMSE and maximum error of position. For velocity, the RMSE east velocity is lowered and improved by 44% and 48.84% compared to the XBOW RISS/GPS and Rad/RISS/GPS systems, respectively. Additionally, the maximum error east velocity is reduced by 38.1% and 51.42% compared to the XBOW RISS/GPS and Rad/RISS/GPS systems, respectively. The RMSE north velocity is also improved by 45.78% and 42.3% compared to the XBOW RISS/GPS and Rad/RISS/GPS systems, respectively. The maximum error north velocity is reduced by 40.15% and 41.18% compared to the XBOW RISS/GPS and Rad/RISS/GPS systems, respectively.

## 6. Conclusions

In conclusion, our research introduces a pioneering navigation system, the XBOW-ANFIS RISS/GNSS, specifically engineered to elevate navigation accuracy and reliability in the face of challenging urban environments characterized by frequent GNSS signal disruptions. The core of our innovative approach lies in the seamless integration of a modified MEMS-IMU and the cutting-edge ML-based ANFIS within a 3D RISS mechanization alongside GNSS.

During extensive real-world trajectory tests encompassing five distinct outage scenarios with varying dynamics, our proposed system demonstrated substantial performance improvements over conventional integrated systems, namely (XBOW-RISS/GPS) and (Rad/RISS/GPS). The tangible outcomes include a noteworthy 54.23% and 51.14% reduction in RMSE azimuth when compared to XBOW RISS/GPS and Rad/RISS/GPS, respectively. Additionally, we observed significant enhancements in 2D/3D position accuracy and various velocity components. The proposed system achieves a 43.8% and 28.2% reduction in the RMSE and maximum error of the 2D/3D position compared to the XBOW RISS/GPS and Rad/RISS/GPS, respectively. Moreover, the system achieves a significant improvement in the reduction percentage of the east velocity by 44% and 48.84% compared to the XBOW RISS/GPS and Rad/RISS/GPS, respectively. The maximum error in the east velocity is reduced by 38.1% and 51.42% compared to the XBOW RISS/GPS and Rad/RISS/GPS, respectively. Additionally, the north velocity is enhanced in the RMSE by 45.78% and 42.3% compared to the XBOW RISS/GPS and Rad/RISS/GPS, respectively. The maximum error in the north velocity is reduced by 40.15% and 41.18% compared to the XBOW RISS/GPS and Rad/RISS/GPS, respectively.

These findings underscore the effectiveness of the XBOW-ANFIS RISS/GPS system, positioning it as a compelling alternative in scenarios demanding precise navigation during GNSS outages. Beyond numerical metrics, the practical applicability of our solution becomes evident, promising enhanced navigation accuracy for autonomous vehicles navigating urban landscapes.

Moreover, the successful integration of ML-based ANFIS with a modified RISS mechanization not only advances the field of navigation but also holds promise for wider applications in autonomous vehicles and related domains. The robust performance demonstrated in real-world tests signifies the XBOW-ANFIS RISS/GPS system as a valuable contribution, ensuring navigation safety and efficiency in urban settings.

In summary, our research not only introduces a novel navigation paradigm supported by empirical evidence but also emphasizes the practical impact and potential applications of the proposed XBOW-ANFIS RISS/GPS system. The comprehensive findings offer a roadmap for future advancements, contributing to the evolving landscape of autonomous vehicle navigation in challenging urban scenarios. 

## Figures and Tables

**Table 1:** Utilized IMUs’ specifications.

IMUs	IMU300CC (XBOW) (100 Hz)	IMU-CPT (100 Hz)
Size (cm^3^)	7.62 × 9.53 × 3.2	15.2 × 16.8 × 8.9
Weight	0.59 kg	2.28 kg
Max data rate	200 Hz	100 Hz
Start-up time	<1 s	<5 s
**Accelerometer**		
Range	±2 g	±10 g
Bias instability	±30 mg	±0.75 mg
Scale factor	<1%, 1σ	300 ppm, 1σ
**Gyroscope**		
Range	±100°/s	±375°/s
Bias instability	<±2.0°/s	±1.0°/h
Scale factor	<1%, 1σ	1500 ppm, 1σ

**Table 2:** 2D/3D position RMSE comparison.

	2D Position RMSE				
Outages no./ Duration	1 (50 s)	2 (90 s)	3 (60 s)	4 (150 s)	5 (70 s)
XBOW RISS/GPS	45.57 m	17.00 m	15.49 m	16.78 m	37.63 m
Rad/RISS/GPS	36.64 m	14.90 m	13.21 m	10.92 m	20.05 m
XBOW-ANFIS RISS/GPS	15.60 m	14.45 m	11.08 m	16.28 m	10.93 m
	**3D Position RMSE**				
XBOW RISS/GPS	45.62 m	17.10 m	15.67 m	16.83 m	37.68 m
Rad/RISS/GPS	36.65 m	14.90 m	13.23 m	10.93 m	20.06 m
XBOW-ANFIS RISS/GPS	15.87 m	14.68 m	11.13 m	16.75 m	11.04 m

**Table 3:** 2D/3D position maximum error comparison.

	2D Position Max Error				
Outages no./ Duration	1 (50 s)	2 (90 s)	3 (60 s)	4 (150 s)	5 (70 s)
XBOW RISS/GPS	68.72 m	34.09 m	23.58 m	29.95 m	67.53 m
Rad/RISS/GPS	54.84 m	23.17 m	19.88 m	23.12 m	32.15 m
XBOW-ANFIS RISS/GPS	25.78 m	27.67 m	15.84 m	29.37 m	18.76 m
	**3D Position Max Error**				
XBOW RISS/GPS	68.75 m	34.14 m	23.70 m	29.97 m	67.56 m
Rad/RISS/GPS	54.85 m	23.17 m	19.90 m	23.13 m	32.52 m
XBOW-ANFIS RISS/GPS	25.95 m	27.79 m	15.87 m	29.63 m	18.82 m

**Table 4:** Overall performance comparison.

	Azimuth (RMSE) (°)	Azimuth (Max Err) (°)	2D Position (RMSE) (m)	2D Position (Max Err) (m)	East Velocity (RMSE) (m/s)	East Velocity (Max Err) (m/s)	North Velocity (RMSE) (m/s)	North Velocity (Max Err) (m/s)
XBOW RISS/GPS	27.18	11.30	26.5	44.77	2.77	7.14	2.49	6.45
Rad/RISS/GPS	25.46	9.80	19.14	30.70	3.03	9.10	2.34	6.69
XBOW-ANFIS RISS/GPS	12.44	5.62	13.67	23.61	1.55	4.42	1.35	3.89

## Data Availability

Data are contained within the article.
